# A Multiphase Coupled Hydrodynamic Model for Fate and Transport Simulation of Polycyclic Aromatic Hydrocarbons in a Semi-Closed Narrow Bay

**DOI:** 10.3390/toxics11070634

**Published:** 2023-07-21

**Authors:** Jiayi Cheng, Ying Wang, Yuxia Li, Lingna Kong, Xiaomeng Wang, Jianbo Han

**Affiliations:** National Marine Environmental Monitoring Center 1, No. 42 Linghe Str., Shahekou District, Dalian 116023, China; jycheng@nmemc.org.cn (J.C.);

**Keywords:** semi-closed narrow bay, PAHs, multiphase coupled, carcinogenic risk assessment, numerical simulation

## Abstract

With their unique geographical characteristics, semi-closed narrow bays are important places for human survival but vulnerable to pollution. Because pollutants (polycyclic aromatic hydrocarbons, PAHs) migrate and undergo transformation through a dynamic mechanism in bays of this type, environmental authorities have formulated a series of effective measures for pollution prevention and control, but these are difficult to realize. Based on monitoring and historical data, a multiphase-coupled hydrodynamic model combined with a carcinogenic risk-assessment model was able to solve the challenging environmental problem. Results showed that the hydrodynamic condition in the semi-closed narrow bay was very complex. A weaker hydrodynamic force had an adverse influence on the diffusion of pollutants, further amplified in part by the head of the semi-closed narrow bay, resulting in a higher ecological risk. The prediction results indicated that the total amount of PAHs transported from seawater to sediments was about 4.7 × 10^13^ ng/year, which might cause serious threats to aquaculture or human health.

## 1. Introduction

With their unique geographical environment, superior natural conditions, and rich marine resources, bays are the best sites for human development and utilization, especially the semi-closed narrow bays [1]. Because of their limited width and extended length, they are ideal places for aquaculture, transport and harbor industries [2]. They play an important role in human survival and developing and utilizing marine resources [3]. However, semi-closed narrow bays also have negative properties [4]. Located in the sea–land interaction area, the substance, energy and physicochemical characteristics of the two systems are integrated closely [5], which makes narrow semi-closed bays very vulnerable to the influence of massive pollutants from the land [6]. The features of narrowness and length cause difficulties in self-purification and diffusion of pollutants [7], creating weak hydrodynamic conditions. Once pollutants are discharged, they will cause long-term pollution which will pose a serious threat to the health and stability of the marine ecosystem [8].

In recent years, the use of fossil fuels and the landfill and incineration of domestic waste have constantly introduced persistent toxic substances or PAHs into the environment in China’s coastal areas [9,10,11]. With the strong teratogenic, carcinogenic, and lethal effects of toxins on the larvae and embryos of algae, shrimp and shellfish [12,13,14], PAHs could seriously endanger the growth, development and reproduction of aquatic organisms [15]. In addition, PAHs are not easy to metabolize and decompose, resulting in biological enrichment through the food chain, which would cause harm to fish, marine mammals and even human beings [3,16]. Consequently, the pollution situation in semi-closed, narrow bays has become one of the most concerning environmental problems to China’s Environmental Authorities [17]. Nevertheless, it is difficult and challenging to formulate a series of effective measures for pollution prevention and control [18,19]. On the one hand, the complex hydrodynamic conditions in a semi-closed narrow bay lead to the inhomogeneous distribution of pollutants in the same medium [20]. The traditional Fugacity Box Model is difficult to put into use; on the other hand, due to the continuous physical and chemical reactions of PAHs in the marine environment, it is impossible to simplify PAHs into a Conservative Pollutants [21].

Based on the experimental and historical analysis results, combined with a numerical simulation calculating method, this study took PAHs as the research object, and the research area (i.e., a semi-closed, narrow bay) was divided into thousands of units by an orthogonal- curve grid-division method [22] connected by hydrodynamics. To simulate the migration and transformation process of PAHs in a practical way, a multiphase-coupled hydrodynamic model was established and combined with air–sea exchange, transportation, deposition, resuspension, adsorption, desorption, degradation and a series of physical and chemical processes [23,24,25].

The paper was devoted to the problem of constructing the fate simulation model of polycyclic aromatic hydrocarbons and presenting the PAH data in the waters and sediments of Pulandian Bay. Refining, deducing and stacking the important information would make it possible to reveal the migration and transformation characteristics of PAHs in the complex, semi-closed narrow bay. Combined with the carcinogenic risk assessment model, a hydrodynamic model was able to solve the challenging environmental problem [26]. Not only the simulation results of PAHs were given, but also the ecological health risk distribution of PAHs was displayed, based on which the Environmental Authority would be able to ascertain the areas that need focus for pollution prevention and control.

## 2. Materials and Methods

### 2.1. General Situations of Study Region

**Figure 1:**
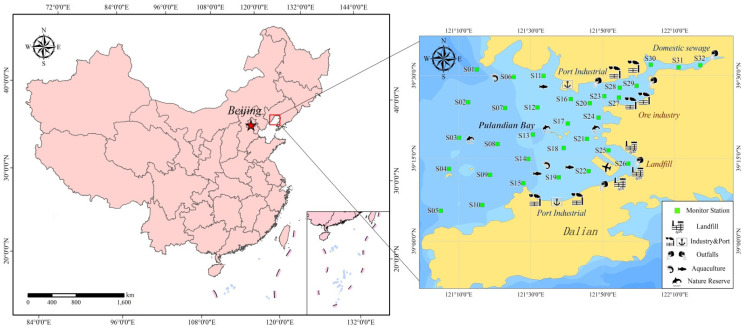
Monitoring stations and study area.

Pulandian Bay is a typical narrow, semi-closed bay located in northeast China (43°00′N–44°00′N, 122°00′E–123°00′E). With an intensive distribution of aquacultures, salt industries, and port industries, Pulandian Bay was chosen as the research area [27]. In addition, as an important supporting hinterland of the cities, many landfill sites have been built along the coast. During the last 20 years, due to continuous intensive industrial activities and sewage discharge, the pollution problem of organic pollutants (primarily, PAHs) in Pulandian Bay had become more and more serious [28]. Therefore, PAHs were chosen as the pollution factor to be researched [5]. The overview of the study region and monitoring stations was shown in Figure 1.

### 2.2. Construction of the Model

**Figure 2:**
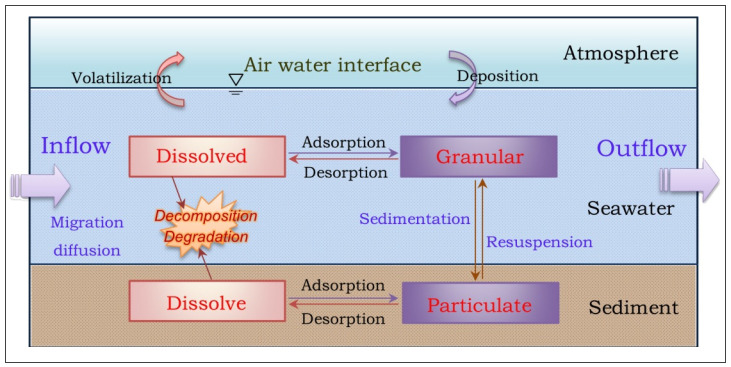
Migration and transformation kinetics process of PAHs.

With the help of Delft3D, a multiphase coupled hydrodynamic model of the PAHs in Pulandian Bay was constructed. The dynamic equation was solved by using the explicit and implicit alternating numerical integration method to simulate the migration and transformation process of PAHs in the marine environment [29,30,31]. Figure 2 is the schematic diagram of the model.

#### 2.2.1. The Primary Equations of the PAHs Dynamic Model

Hydrodynamic equations:

(1) $$\frac{\partial \zeta }{\partial t}+\frac{\partial \left(HU\right)}{\partial X}+\frac{\partial \left(HU\right)}{\partial Y}=0$$

(2) $$\frac{\partial U}{\partial t}+U\frac{\partial U}{\partial X}+V\frac{\partial U}{\partial Y}-fV+g(\frac{\partial \zeta }{\partial X}+\frac{U\sqrt{U^{2}+V^{2}}}{H\psi^{2}})=\frac{1}{\rho_{0}}(\tau_{vx}-\psi )$$

(3) $$\frac{\partial V}{\partial t}+U\frac{\partial V}{\partial X}+V\frac{\partial V}{\partial Y}-fU+g(\frac{\partial \zeta }{\partial Y}+\frac{V\sqrt{U^{2}+V^{2}}}{H\psi^{2}})=\frac{1}{\rho_{0}}(\tau_{vy}-\psi )$$

(4) $$\tau =C_{D}\rho_{A}W^{-2}$$

PAHs Kinetic diffusion:

(5) $$\frac{\partial C_{i}}{\partial t}+\frac{\partial }{\partial X}(\frac{V_{e}\partial C_{i}}{\sigma_{tx}\partial X})+\frac{\partial }{\partial Y}(\frac{V_{e}\partial C_{i}}{\sigma_{ty}\partial Y})=\frac{\partial \left(UC_{i}\right)}{\partial X}+\frac{\partial \left(VC_{i}\right)}{\partial Y}+Q$$

Exchange dynamics of PAHs between seawater and atmosphere:

(6) $$R_{vol}=\frac{k_{vol}\times \left(C_{i}-C_{equ}\right)}{H}$$

(7) $$k_{vol}=1/(\frac{1}{k_{l}}+\frac{R_{g}\left(Tem+273.15\right)}{N_{g}Pk_{g}e^{\left(a_{1}+a_{2}/\left(Tem+273.15\right)\right)}})$$

Exchange dynamics of PAHs between seawater and seabed:

(8) $$R_{p}=v_{p}\times C_{i}$$

(9) $$C_{i}=\left\{\begin{array}{c} C_{sed}=C_{par}+C_{d},\;\;if\;v_{p}\geq 0\\ C_{w}=C_{par}+C_{d\;},\;\;if\;v_{p}\leq 0 \end{array}\right.$$

(10) $$v_{p}{=\varphi \;\times \;D}_{sw}\times \frac{C_{sed}-C_{s}}{L_{sw}}$$

Comprehensive decomposition (degradation) process:

(11) $$R=K_{0}+K_{1}\times K_{t}\times C_{i}$$

Stokes formula (deposition):

(12) $$V_{0}=\omega \;\times \frac{\rho_{T}-\rho_{0}}{V_{e}}{\;\times \;g\;\times \;d}^{2}$$

where a_1_ was the temperature coefficient for volatization entropy, a_2_ was the temperature coefficient for volatilization enthalpy, C_D_ was the coefficient of wind-stress, *C_i_, C_equ_*, *C_sed_*, *C_w_*, *C_par_*, and *C_d_* were the total concentration, equilibrium concentration, sediment concentration, concentration in upper overlying water, particle adsorption state concentration, and free dissolved state concentration of PAHs, d was the size of suspended particles, *D_sw_* was the diffusion coefficient, f was the Coriolis parameter, g was the acceleration of gravity, H was the water depth, *K_0_* was the zero-order reaction constant, *K_1_* was the first-order reaction constant, *K_t_* was the thermodynamic constant, k_l_ was the transfer coefficient for the liquid film, k_g_ was the transfer coefficient for the gas film, k_vol_ was the seawater-air exchange coefficient, *L_sw_* was the thickness of the overlying water layer, Ng was the number of moles in a m^3^ gas, P was the atmospheric pressure, Q was the pollutant discharge intensity, R was the decomposition rate of PAHs, R_D_ was the diffusion flux, R_g_ was the gas constant, R_vol_ was the seawater-air exchange rate, R_p_ was the permeation flux, t was time, Tem was the ambient temperature, U,V were the velocity components in the X,Y directions, V_0_ was the PAHs sedimentation rate, V_e_ was the seawater viscosity coefficient, v_p_ seepage velocity, W was the wind-speed at 10m above water surface, σtx and σty were the turbulent diffusion coefficient in X and Y direction, ζ was the water level, ψ was the Chézy coefficient, τ (including τ_x_, τ_y_) was wind-stress, φ was the sediment porosity, ω was the concentration of adsorbed PAHs, ρ_0_ was the density of sea water, and ρ_T_ was the density of suspended particles.

#### 2.2.2. Toxicity Analysis Model of PAHs

By ingestion and dermal uptake, PAHs are able to enter the body and spread to various organs, such as the brain, muscles, fat, liver, and kidneys, through alveoli, the digestive system, and blood [17,26]. Therefore, the US Environmental Protection Agency method [32] was used to estimate incremental lifetime cancer risks (ILCRs) to assess potential health risks to humans posed by PAHs.

The ILCRs for the ingestion and dermal uptake were calculated using the equations above [33,34].

**Figure 3:**
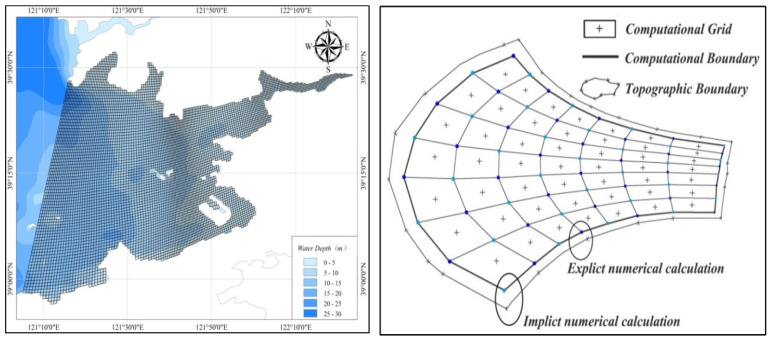
Grid of calculated geometry.

Considering that PAHs are a mixture with many components, many physical or chemical differences exist. To ensure the consistency of PAHs simulation results, the toxicity-equivalent-concentration method was used in this study [35]. For toxicity-equivalent factors of 16 PAHs, see Table 1. The total concentration of 16 PAHs was replaced by a Benzo(a)pyrene toxicity-equivalent concentration.

(13) $$\Sigma PAHs=\sum C_{i}\times TEF_{i}$$

(14) $$ILCR_{ingestion}=\frac{CS_{ingestion}\times \sqrt[{3}]{BW/70\;}\times IR_{ingestion}\times EF\times ED}{BW\times AT\times 10^{6}}$$

(15) $$ILCR_{dermal}=\frac{CS_{dermal}\times \sqrt[{3}]{BW/70\;}\times SA\times AF\times ABS\times EF\times ED}{BW\times AT\times 10^{6}}$$

where *C_i_* was the concentration of individual species of PAHs in a calculation grid (in Figure 3), *TEF_i_* was the toxicity-equivalent factor of individual species of PAHs (Table 1.), ΣPAHs was the total toxicity equivalent of 16 PAHs relative to BAP (ng/g). ABS was the dermal adsorption fraction, AF was the dermal adherence factor (mg/(cm^2^∙h)), AT was the average life span (y), BW was the average body weight (kg), C_S_ was the ΣPAHs concentration (ng/g) in sediment (i.e., ΣPAHs), C_SF_ was the carcinogenic slope factor for ingestion or dermal uptake (mg/(kg∙d)), ED was exposure duration (y), EF was exposure frequency (d/y), IR_Ingestion_ was the intake rate (mg/d), and SA was the dermal exposure area (cm^2^).

The ILCR method mentioned above was used to evaluate the health risk caused by PAHs. The ILCR parameters used in the equation are shown in Table 1 and Table 2.

(16) $$\sum ILCR=ILCR_{ingestion}+ILCR_{dermal}$$

The ILCR value was divided into three levels, according to the U.S. Environmental Protection Agency’s guidelines on health risk assessment of PAHs. When the ILCR value < 10^–6^, it indicates that the health risk is low or negligible; when the ILCR value is between 10^–6^–10^–4^, it indicates a potential carcinogenic risk; when the ILCR value >10^–4^, it indicates a high carcinogenic risk.

### 2.3. Model Parameter Setting

The boundary of the study area was extracted from the satellite remote sensing image. ArcGIS (10.3, ESRI) was applied to vectorize the terrain, water depth, and boundary and determine the open and closed boundary as well as the calculation domain. The dynamic process of pollutants in a narrow and semi-closed bay was so complex that orthogonal curved grids were used, which could be closer to the terrain boundary. To cover every small corner of the complex topography of Pulandian Bay, the whole calculation domain was compartmentalized into 32,332 (274 × 118) orthogonal curved grids (Figure 3).

The water depth in Pulandian Bay was shallow, ranging from 5 to 25 m, and the topographic relief was smooth and level. A two-dimensional ocean hydrodynamics model was suitable. The model-time step was set as 5.0 min. Gravity, geostrophic deflection force, and tidal effect were the main driving forces of the model. The gravity acceleration g was 9.81 m/s^2^, the seawater density was 1024.2 kg/m^3^, the diffusion and viscosity coefficients were 10.1m^2^/s and 1.0 × 10^−6^ m^2^/s. Tidal harmonic constants were obtained from the harmonic analysis based on 2015 tidal data (including, K_1_, M_2_, O_1_, S_2_, P_1_, Q_1_, M_1_, N_2_, K_2_). The primary parameters of the model are shown in Table 3.

### 2.4. Initial Conditions

The dynamic process of pollutants in the narrow and semi-closed bay was very complex. The hydrodynamics force, the physical and chemical reactions of air–seawater, seawater–sediment, and the degradation of pollutants themselves lead to large fluctuations in their equilibrium state in different media (such as air, water, or sediment). To ensure the accuracy of the numerical calculation model simulation, the calculation scheme of “hot start” was implemented instead of “cold start.” Therefore, the initial concentration of PAHs in Pulandian Bay was set as ϛ_i_ and ξ (according to the monitoring results of PAHs).

(17) $${ϛ}_{\mathrm{PAHs}}={\left.f\left(x,y,t\right)\right|}_{t=0}=\left\{\begin{array}{c} {ϛ}_{i},\;\;if\;\left(x,y\right)\in \;{∯}_{1}^{n}d\theta \\ \xi ,\;\;if\;\left(x,y\right)\notin \;{∯}_{1}^{n}d\theta \end{array}\right.$$

The pollution source intensity in the model showed the equivalent average value of 120 μg/L·(s^−1^m^3^) [28]. The position of the discharge sources is shown in Figure 1. Once the simulation started, a series of migration and transformation processes of PAHs, such as atmosphere–seawater exchange, seawater–sediment exchange, and sedimentation and degradation began. Synchronously, the mixing process of seawater both inside and outside the bay occurred, which was the dominant function for contaminant diffusion.

### 2.5. Accuracy Verification of the Model

To ensure the multiphase coupled hydrodynamic model could effectively simulate the fate and transport of PAHs in Pulandian Bay, accuracy verification was critical. Due to the massive amount and complexity of calculation, not only a strong mathematical and computer programming ability was required, but also a large number of long-term historical data needed to be used for calibrating the model parameters and improving its accuracy. In this study, three aspects were taken into consideration for the accuracy verification of the model. The first was to ensure the accuracy of the on-site monitoring data of PAHs and hydrology; the second was to calibrate the model parameters during the process of model construction; the third was to verify the accuracy of the simulation results after the calculation.

#### 2.5.1. Collection and Experimental Analysis

In the summer of 2020, the investigation and sampling of hydrology, water quality and sediment in Pulandian Bay through a field survey were carried out. A total of 32 monitoring stations were set up. To verify the accuracy of the model simulation, three water-quality monitoring stations, three hydrological-monitoring stations (S3, S18 and S20), and two tide-level observation stations (S11 and S15) were set up. The layout of the sampling stations is shown in Figure 1. A grab sampler (iron) was used to get the surface sediment (0–10 cm) samples, and a layered water sampler (stainless steel, 5 L) was used to get seawater samples. Hydrologic observations (flow velocity and direction) were collected every 10 min by using an Acoustic Doppler Current Profiler (Model: WHS600, depth unit size: 600 kHz).

Seawater samples were enriched by an envi-C18 solid phase membrane disk (flow rate: 12 mL/min−30 mL/min, vacuum filtration) extraction, wrapped in aluminum foil, sealed, and stored before testing (SPE samples). Sediment samples were freeze-dried (vacuum freeze dryer, 24 h) and ground (180 μm) after removal of impurities (e.g., plant residues and animal). Brown glass bottles were used for storage and then brought back for analysis.

Sediment samples were weighed for about 20.0 g and then the following were added: anhydrous sodium sulfate 20.0 g, copper powder 20 g, 100 mL of extraction solution [acetone:n-hexane (V:V) = 1:1] and recovery indicators (D8-p, p’-DDT, CB155, CB65 and TBB). The samples were reflux-extracted through a Soxhlet apparatus for 24 h. The extract was evaporated to about 2 mL by rotary evaporation and stored. Water samples (SPE samples) were extracted with dichloromethane (3 times, 5 mL/time) and n-hexane (2 times, 5 mL/time), respectively, and the extract was concentrated to about 2.0 mL for further purification. Samples were purified by chromatography on silica gel/alumina columns. N-hexane (80 mL) and 7:3 *v*/*v* dichloromethane/n-hexane (100 mL) were used. The eluate was concentrated to 200 μL by high-purity nitrogen and spiked with terphenyl-d14 (10 μL × 10.0 μg/mL) as the internal standard. Spiked samples were stored at the temperature of −20 °C until analysis.

Sixteen PAHs listed as priority chemicals by the US EPA (*Metabolism of Polycyclic Aromatic Hydrocarbons in the Environment*) were analyzed by gas chromatography-mass spectrometry (GC6890N/MSD5975B, Agilent Co., USA) on a DB-5ms capillary column (30 m × 0.25 mm × 0.25 μm, Agilent Co., USA). The electron impact ionization source was set to 230 °C, and the quadrupole and transmission line was 150 °C and 290 °C. Selected ion monitoring was performed and the scanning range was M/Z 0–500 amu. Of all samples and blanks, the range of recovery rates of D8-p, p’-DDT, CB155, CB65 and TBB were 47.3–78.1% (54.6%), 62.5–92.4% (73.7%), 75.1–94.6% (83.8%), 83.2–103.9% (89.5%), and 84.7–111.6% (92.4%), respectively. All data had subtracted the blank. The limit of detection was set as 10 times the standard deviation of the spiked minimum-concentration blank (10 × SD). Assuming a concentration volume of 200 μL, the LOD was 0.02 ng/L for seawater and 0.05 ng/g (d.w.) for sediments.

#### 2.5.2. Model Parameter Calibration

To improve the accuracy of the PAH numerical calculation model, the historical data (1999~2019) of seawater PAHs, which was monitored by the Dalian Marine Environment Monitoring Center Station of the State Oceanic Administration in Pulandian Bay, were used to calibrate the model parameters. The calibrated model parameters included the seawater viscosity coefficient (V_e_), the transfer coefficient for the liquid or gas film (k_l_, k_g_), the diffusion coefficient (D_sw_), and the Chézy coefficient (Ψ).

Station S3 was used for model parameter calibration. The concentration of PAHs in seawater was taken as the judgment basis for the accuracy of model calibration results. According to the existing research [36], although the specific values of the above 5 parameters cannot be formulated directly, they are controlled by multiple factors such as topography and water depth. However, the value range of parameters can be estimated. Therefore, based on the existing research experience [37], the value range of each parameter was preliminarily set as V_e_ ∈ [1.44, 1.57], D_sw_ ∈ [0.12, 0.15], Ψ ∈ [0.02, 0.06], k_g_ ∈ [0.73, 0.78], k_l_ ∈ [4.12, 4.19].

**Figure 4:**
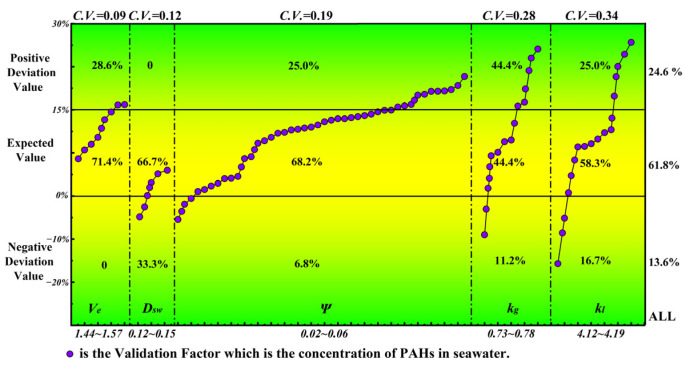
Parameter calibration process of numerical calculation model.

In the process of parameter calibration, we found that the value of the seawater viscosity coefficient (V_e_) had a strong impact on the concentration of PAHs in seawater simulated by the model [38]. Therefore, we encrypted the calibration of V_e_ parameters (raised to 48 times). When the numerical simulation results of PAHs were basically consistent with their measured concentrations (in seawater), this indicated that the results of the model parameters were reasonable and the calibration work was completed. (See Figure 4 for further information). The parameter calibration results are shown in the Table 4 below.

**Figure 5:**
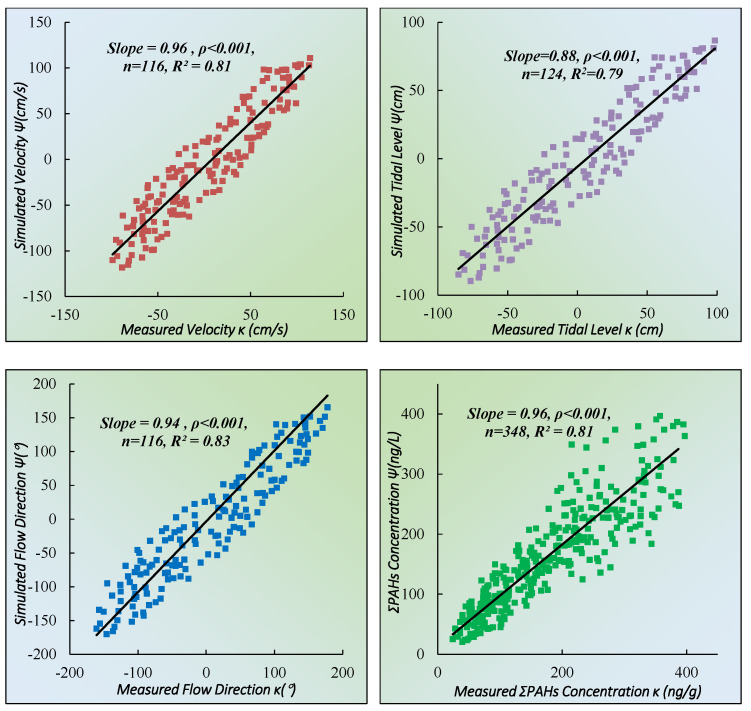
Accuracy of parameter calibration results.

To ensure the accuracy of the calibration results of model parameters, after determining the value of Ve, Dsw, Ψ, kg, and kl, the historical data (1999~2019) of seawater PAHs and hydrology data (including the tidal level, flow velocity and direction) were used to verify. The verification results showed that the predicted result of thePAHs numerical calculation model was in great agreement with the monitoring value, which proved that the model parameters were set appropriately (Figure 5).

#### 2.5.3. Model Accuracy Verification

**Figure 6:**
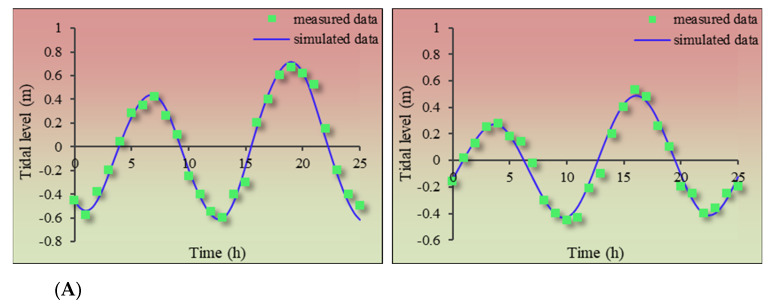

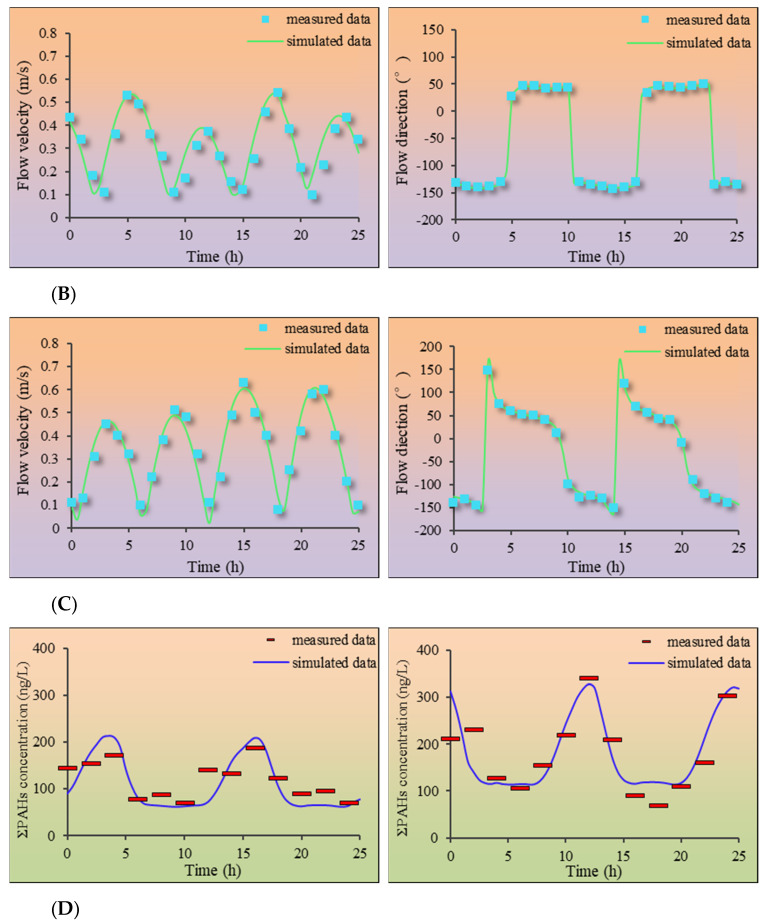
Verification results of water level, flow velocity and direction and ΣPAHs concentration. (**A**) is the verification results of water level in station S11 and S15, (**B**) is the verification results of flow velocity and flow direction in station S3, (**C**) is the verification results of flow velocity and flow direction in station S20, (**D**) is the verification results of ΣPAHs concentration in station S3 and S18.

To ensure the accuracy of the numerical simulation results, the numerical simulation model was verified based on the measured data (in 2020) of tide level, flow velocity, flow direction and ΣPAHs concentration in seawater continuously observed for 25 h at quality control stations (more information in Section 2.5.1). The results of model accuracy verification are shown in Figure 6.

For further verification, the statistical method proposed by Willmott (1981) [39] was used to evaluate the model:

(18) $$\mathrm{Skill}=1\;-\;\frac{\sum_{i=1}^{n}/M_{i}-D_{i}{/}^{2}}{\sum_{i=1}^{n}(/M_{i}-\overset{-}{D}/+/D_{i}-\overset{-}{D}/{)}^{2}}$$

where D_i_ represented the observed value; $\overset{-}{D}$ represented the observed average value; i = 1, 2, …, n; Mi represented the simulated value; N represented the number of water samples; Skill represented the degree of correlation between the deviation of the observed value and observed average value and the deviation of the simulated value and the observed average value.

The model’s performance was rated according to the Skill value: <0.2 was poor; 0.2–0.5 was medium; 0.5–0.7 was good; >0.7 was excellent. According to the verification results, the accuracy of the model was satisfactory: 0.83–0.88 for tide level; 0.81–0.84 for flow velocity; 0.85–0.89 for flow direction, and 0.77–0.83 for PAHs concentration in seawater indicated that the numerical hydrodynamic model was convincing and reliable.

## 3. Results and Discussion

### 3.1. Hydrodynamics Simulation Results

**Figure 7:**
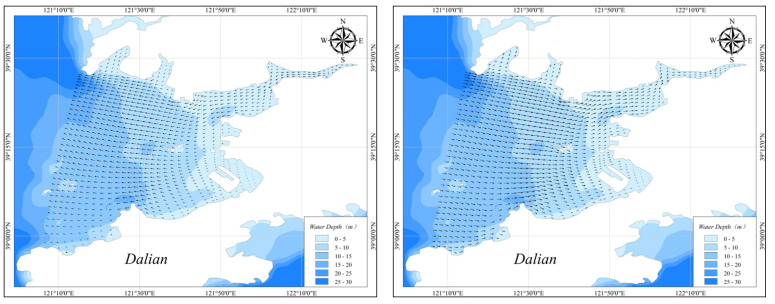
Simulation of hydrodynamics during rising or falling tide.

The hydrodynamics simulation results for Pulandian Bay are shown in Figure 7. The arrows denote the flow direction, and length is proportional to the flow velocity. The simulation results showed that the hydrodynamic conditions in Pulandian Bay were complex. From the bay-head to the bay-mouth, Pulandian Bay could be divided into two parts. With a high velocity, the bay-mouth area is relatively wide, and the flow direction is evenly and uniformly distributed; however, the bay-head area is narrow with a lower velocity, where the flow direction varies greatly and is distributed turbulently. In general, the hydrodynamics here had significant spatial distribution characteristics: the closer to the bay-head, the weaker the hydrodynamics.

### 3.2. The Simulation Results of PAHs

**Figure 8:**
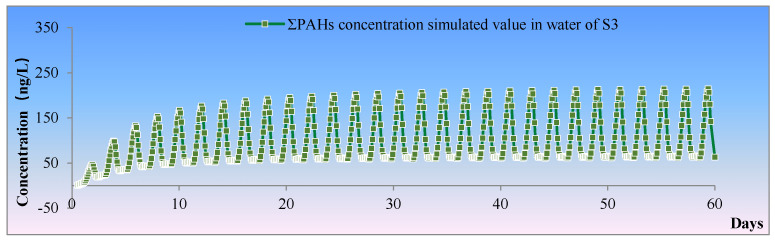

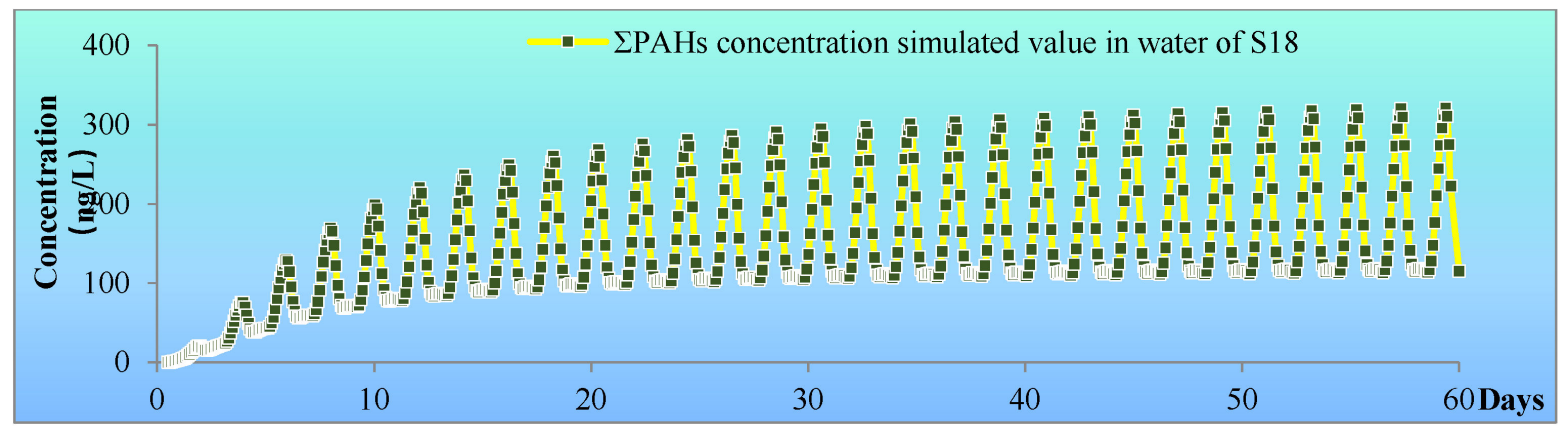
Simulation of the ΣPAHs concentrations in S_3_ and S_18_.

Time-series characteristic analysis: To explore the variation characteristics of ΣPAHs’ concentration with time in Pulandian Bay, the model of PAHs was used to simulate the migration, transformation, and sedimentation process. The variation of PAHs simulated concentration with time at the bay-head (S20) and the bay-mouth (S3) was extracted from the model, and the concentration variation curve was drawn in Figure 8. From the curves, it could be learnt that with the simulated time going by, the concentration of ΣPAHs increased with fluctuations and finally reached a dynamic equilibrium (i.e., the concentration of PAHs fluctuates within a certain range).

The concentration of ΣPAHs arrived at a stable level after 20 days (about 40 tidal cycles) at S3. However, the concentration of ΣPAHs did not reach dynamic equilibrium until 50 days (100 tidal cycles) at S20.

S3 was at the bay-mouth of Pulandian Bay, which had stronger hydrodynamics and ample water exchange. Consequently, the concentration of ΣPAHs at S3 was able to arrive at a dynamic equilibrium quickly, after only 20 days (40 tidal cycles). In contrast, the hydrodynamics of S20 was poor, located at the bay-head, where the ΣPAHs concentration kept increasing even after 40 days (80 tidal cycles). It seemed that this phenomenon was ordinary, but it was of great significance. The closer the offshore distance, the more complex were the hydrodynamic conditions and the weaker was the water-exchange capacity. Therefore, the diffusion of pollutants was more unfavorable. On the contrary, with a farther offshore distance, the hydrodynamic conditions would be stronger, which would be conducive to the rapid diffusion and dilution of pollutants.

It could be seen that the concentration of pollutants in the marine environment changed periodically, often due to tidal fluctuations. First-hand observation made it clear why it is difficult to get the real concentration of a pollutant in the marine environment through one-time monitoring, which was the largest problem for staff who had been engaged in marine environmental monitoring for a long time [40]. The migration and transformation process of pollutants in the marine environment is strongly affected by the hydrodynamics, which will be expanded further, especially in the bay or estuarine area. Time series monitoring is usually more convincing than spatial, but, due to the shortage of funds, manpower or equipment, it is very difficult and costly to implement regular monitoring at sea. Therefore, in order to explore the diffusion characteristic of pollutants and understand the tendencies of marine environmental quality, taking advantage of a numerical calculation model may be a more effective means.

Analysis of spatial distribution characteristics: After numerical simulation was started, the water in the calculation domain would exchange with the external to complete the PAHs transportation and migration. Specifically, with the tide rising, the external water (clean) flowed into the bay and mixed with the water init, resulting in the decreased concentration of ΣPAHs. After a certain time, the water exchange would be further completed towards the inner area of the bay. At ebb tide, the water with higher PAHs concentration in the bay would diffuse outside to complete the transportation and transfer. When the process of the next tidal cycle began, the cycle would be repeated.

**Figure 9:**
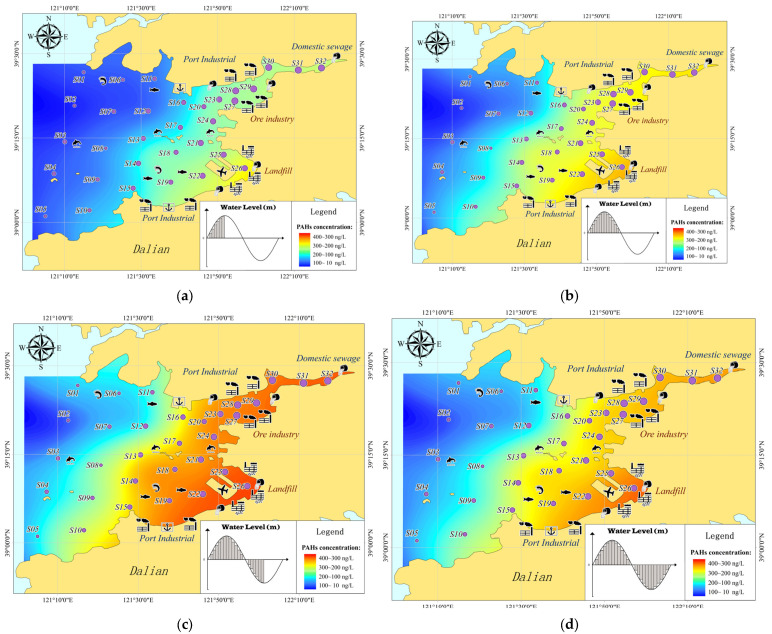
Simulation of the ΣPAHs concentration distribution in the seawater: (**a**) *High Tide* (**b**) *Falling Tide* (**c**) *Low Tide* (**d**) *Rising Tide*.

Figure 9 shows the concentration distribution of PAHs in high tide, falling tide, low tide, and rising tide during the numerical simulation, showing the spatial evolution process of PAHs migration and diffusion in Pulandian Bay. From the perspective of spatial distribution, the PAHs completed a water exchange from inside to outside under the force of hydrodynamics. The diffusion rate of PAHs varied significantly with the distance from the bay-head. Generally, the phenomenon showed a distribution trend of strengthening the hydrodynamic from the bay-head to the bay-Mouth. PAHs were able to stay in the bay-head area, where the hydrodynamic conditions were poor and the water exchange capacity was insufficient, that for an especially long time.

### 3.3. Analysis of PAHs in Seawater-Sediment Exchange

**Figure 10:**
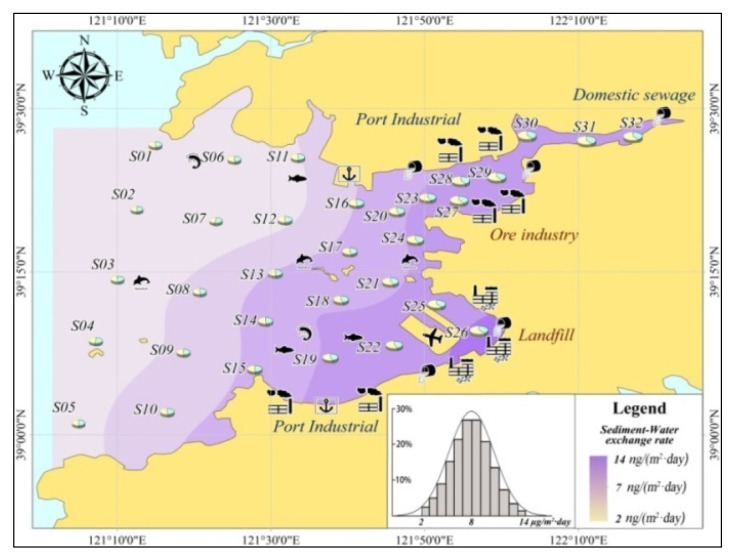
Simulation of the ΣPAHs sedimentation rate.

As described above, PAHs in the marine environment were easily adsorbed on suspended particulate materials and migrated with them. Therefore, when the suspended solids settled, the PAHs would also deposit and become part of the sediments. In order to explore the exchange characteristics of PAHs between seawater and sediment, based on the numerical simulation-calculating results, the distribution of seawater–sediment exchange rate of PAHs is illustrated (Figure 10).

Figure 10 shows that the sedimentation of PAHs was the primary process of seawater–sediment exchange in Pulandian Bay, where the PAHs mainly migrated and transformed from seawater into sediment. Spatially, the results of the numerical simulations showed that the sedimentation rate exhibited an increasing distribution tendency from the bay-mouth (2 ng/m^2^·day) to the bay-head (14 ng/m^2^·day) in Pulandian Bay. According to the statistics of normal distribution, the sedimentation rate of PAHs (more than 60% grid units) was generally between 6–10 ng/m^2^·day.

Compared with other bays (Guanabara Bay, Fraser River Basin e.g.,) in the world, the sedimentation rate of the PAHs was slightly high. After superposition, the total amount of PAHs transported from seawater to sediments every year was about 4.7 × 10^13^ ng in Pulandian Bay. This might cause serious threats to aquaculture and human activities in nearshore waters. Because of the strong bioaccumulation function of PAHs, they might eventually cause damage to human health decades later. Therefore, in the following section, based on the monitoring data of PAHs, the numerical calculation model was coupled with the health risk assessment model so as to explore the extent to which pollution levels of PAHs in Pulandian Bay pose a risk to human health.

### 3.4. Health Risk Assessment of PAHs

As mentioned above, a single monitoring alongside survey results was not comprehensive enough, both temporally and spatially. However, most of the pollutant health risk assessment models were based on the identified and single survey/monitoring data results, which would inevitably introduce error and randomness. Therefore, to solve this difficulty, a dynamic model of health risk assessment for PAHs was built by coupling the health risk assessment model with the numerical calculation model. Depending on model parameter calibration and validation, it was possible to minimize the occasionality and uncertainty due to sampling and analysis processes, which could improve the authenticity and accuracy of evaluated conclusions.

According to the calculation results, Pulandian Bay ΣILCR values were 2.8 × 10^−9^~4.6 × 10^−6^ (some areas were larger than 10^−6^). Thus, PAHs in the bay-head of Pulandian could pose a potential threat to human health, and their carcinogenic risk index ΣILCR was higher than that at the bay-mouth. Specifically, the ILCR_ingestion_ was 10^−8^–10^−6^; the ILCR_dermal_ was 10^−9^–10^−6^.

**Figure 11:**
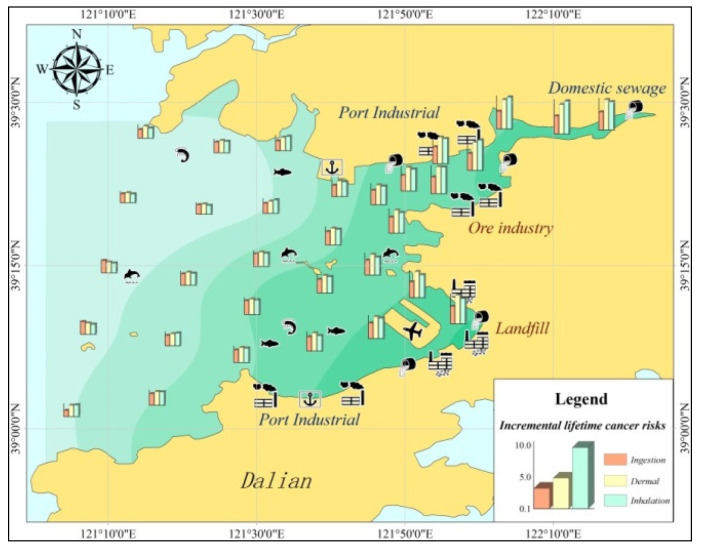
Health risk assessment results of PAHs in Pulandian Bay.

As shown in Figure 11, the distribution of aquaculture in the Pulandian Bay was extremely intensive, especially in the nearshore waters, where the health risks of PAHs were so high that they could cause toxic effects on aquatic life or even pose a threat to human health through bioaccumulation. In this paper, it is suggested that the Marine Environmental Authority should pay more attention to control efforts to prevent PAH pollution in the head of Pulandian Bay. In order to minimize the pollution and impacts of PAHs on nearshore waters, monitoring amounts of PAHs pollutants or reference to the Pollutant Discharge Permit System might be an effective new measure [40].

## 4. Conclusions

The hydrodynamic conditions in the semi-closed, narrow Pulandian Bay were very complex and showed the conspicuous spatial distribution characteristics that the closer to the bay-head, the weaker the hydrodynamics.

The concentration of pollutants (PAHs) in the ocean fluctuated periodically with the tide, showing that hydrodynamic force had a significant impact on the diffusion of pollutants. The diffusion rate of pollutants in the area was faster with a stronger hydrodynamic force, and vice versa. This influence would be further amplified in the semi-closed narrow bay, which would lead to a lack of sufficient diffusion and dilution of pollutants in the bay-head area, resulting in a higher ecological risk.

The total amount of PAHs transported from seawater to sediments was 4.7 × 10^13^ ng/year in Pulandian Bay. Arguably, it might cause serious threats to aquaculture and human activities in nearshore waters. Because of the strong bioaccumulation function of PAHs, the threats and damage may eventually be transferred to human health, decades later.

## Figures and Tables

**Table 1:** Toxicity equivalence factors of 16 PAHs.

PAHs	*TEF_i_*	PAHs	*TEF_i_*
Naphtalene (NAP)	0.001	Benzo[a]anthracene BaA	0.1
Acenaphthylene (ANY)	0.001	Chrysene (CHR)	0.001
Acenaphthene (ANA)	0.001	Benzo[b]fluoranthene (BbF)	0.1
Fluorene (FLU)	0.001	Benzo[k]fluoranthene (BkF)	0.1
Fenanthrene (PHE)	0.001	Benzo[a]pyrene (BaP)	1
Anthracene (ANT)	0.01	Indeno [1,2,3-cd]pyrene (IcdP)	0.1
Fluoranthene (FLT)	0.001	Dibenzo[a,h]anthracene (DBA)	1
Pyrene (PYR)	0.001	Benzo[g,h,i]perylene (BPE)	0.01

**Table 2:** Parameters in ILCR model.

Parameters	Unit	Value	Reference
EF	d/year	350	[31]
ED	Year	40	[31]
IR_Ingestion_	mg/d	100	[31]
SA	cm^2^/d	5700	[31]
AF	mg/cm^2^	0.07	[31]
ABS	unitless	0.13	[31]
AT	d	70 × 365	[31]
PEF	m^3^/kg	1.36 × 10^9^	[31]
CS_ingestion_	mg/(kg·d)	7.3	[32]
CS_dermal_	mg/(kg·d)	3.85	[32]
BW	kg	70	[32]

**Table 3:** Parameters of PAHs dynamics Model.

Parameters	Unit	Value	Parameter	Unit	Value
Zero(th) Order Reaction Constant (In Previous Studies, Cheng, 2021)	mol/(L·s)	0.003	Percentage of Organics Adsorbed (In Previous Studies, Cheng, 2021)	%	30.0
First Order Reaction Constant (In Previous Studies, Cheng, 2021)	d^−1^	0.017	Percentage of Organism Adsorbed (In Previous Studies, Cheng, 2021)	%	15.0
Volatilization Diffusion Flux (In Previous Studies, Cheng, 2021)	μg/(m^2^·d)	0.723	Percentage of Water Dissolved (In Previous Studies, Cheng, 2021)	%	10.0
Atmospheric Deposition Flux (In Previous Studies, Cheng, 2021)	μg/d	4.126	Percentage of Particulates Adsorbed (In Previous Studies, Cheng, 2021)	%	45.0
Transfusion Flux (In Previous Studies, Cheng, 2021)	m^2^	0.013	Totally	%	100.0

**Table 4:** Calibration results of model parameters.

Parameter	Symbol	Unit	Calibration Frequency	Value Range	Parameter Value
The Seawater Viscosity Coefficient	*V_e_*	m^2^/s	7	1.44~1.57	1.50
The Chézy Coefficient	*Ψ*	m	48	0.02~0.06	0.035
Transfer Coefficient For the Gas Film	*k_g_*	μg/(m^2^·d)	9	0.73~0.78	0.76
Transfer Coefficient For the Liquid Film	*k_l_*	μg/(m^2^·d)	12	4.12~4.19	4.15
The Diffusion Coefficient Between Water-Seabed	*D_sw_*	μg/(m^2^·d)	3	0.012~0.015	0.014

## Data Availability

Not applicable.
